# Effect of different drugs and drug combinations on killing stationary phase and biofilms recovered cells of *Bartonella henselae* in vitro

**DOI:** 10.1186/s12866-020-01777-9

**Published:** 2020-04-10

**Authors:** Xiaoyan Zheng, Xiao Ma, Tingting Li, Wanliang Shi, Ying Zhang

**Affiliations:** 1 Beijing Tropical Medicine Research Institute, Beijing Friendship Hospital, Capital Medical University, Beijing, 100050 P.R. China; 2 Department of Molecular Microbiology and Immunology, Bloomberg School of Public Health, Johns Hopkins University, Baltimore, MD 21205 USA

**Keywords:** *Bartonella henselae*, Stationary phase, Biofilm, Antimicrobial activity, Drug combination

## Abstract

**Background:**

*Bartonella henselae* is a Gram-negative bacterium transmitted to humans by a scratch from cat in the presence of ectoparasites. Humans infected with *B. henselae* can result in various clinical diseases including local lymphadenopathy and more serious systemic disease such as persistent bacteremia and endocarditis. The current treatment of persistent *B. henselae* infections is not very effective and remains a challenge. To find more effective treatments for persistent and biofilm *Bartonella* infections, in this study, we evaluated a panel of drugs and drug combinations based on the current treatment and also promising hits identified from a recent drug screen against stationary phase and biofilm recovered cells of *B. henselae*.

**Results:**

We evaluated 14 antibiotics and 25 antibiotic combinations for activity against stationary phase *B. henselae* (all antibiotics were at 5 μg/ml) and found that ciprofloxacin, gentamicin, and nitrofurantoin were the most active agents, while clofazimine and miconazole had poor activity. Drug combinations azithromycin/ciprofloxacin, azithromycin/methylene blue, rifampin/ciprofloxacin, and rifampin/methylene blue could rapidly kill stationary phase *B. henselae* with no detectable CFU after 1-day exposure. Methylene blue and rifampin were the most active agents against the biofilm *B. henselae* after 6 days of drug exposure. Antibiotic combinations (azithromycin/ciprofloxacin, azithromycin/methylene blue, rifampin/ciprofloxacin, rifampin/methylene blue) completely eradicated the biofilm *B. henselae* after treatment for 6 days.

**Conclusions:**

These findings may facilitate development of more effective treatment of persistent *Bartonella* infections in the future.

## Background

*Bartonella* species are fastidious, Gram-negative intracellular bacteria [1] that are widely present in various mammals including cats, rodents, ruminants, and humans [2, 3]. They are transmitted mainly by direct contact such as animal scratches and bites, or by some arthropods such as sand flies, lice, fleas, biting flies, and ticks [4]. So far, at least 40 species or subspecies of *Bartonella* have been discovered [5]. At least 13 *Bartonella* species or subspecies are zoonotic [2]. Three species of *Bartonella* including *B. henselae, B. quintana,* and *B. bacilliformis,* are responsible for the great majority of infections in humans [6]. While *B. henselae* is the most common zoonotic *Bartonella* species, and the infection has a worldwide distribution [7] . According to a study from the USA, the incidence of *B. henselae* infection in human is about 3.7 per 100,000 [8]. Cat is its native host, and it is transmitted by the cat flea [9] . However, *B. henselae* can infect humans through an infected cat’s scratch causing cat scratch disease (CSD), which is a disease characterized by self-limiting lymphadenopathy [10]. In the United States, CSD affects about 24,000 people annually [11]. Humans infected with *B. henselae* may also have other various clinical symptoms, such as fever with bacteremia, bacillary peliosis, bacillary angiomatosis and some infected individuals may get life-threatening blood-culture-negative endocarditis (BCNE) [12, 13]. Because *B. henselae* is capable of growing as aggregates and forming biofilms on infected native and prosthetic heart valves, it is a common cause of blood-culture negative endocarditis [14]. *B. henselae* biofilms have been involved in two distinct parts of the life cycle. First, they colonize and persist in the arthropod vector which increases transmission from the flea to the vertebrate host [15]. Second, *B. henselae* biofilms are an important composition of the heart valve vegetations found in patients with BCNE [16]. Biofilms are characterized by their stability, increased resistance to antibiotics, and chronic bacterial infections. In addition, biofilms protect the bacteria from antibiotics and host immune defenses such as macrophage engulfment [17]. Studies have shown that treatment failures of *Bartonella* infections are a significant problem despite low MICs suggesting a persistence problem [18]. *B. henselae* has a substantial capacity to withstand antimicrobial agents due to bacterial persistence and biofilm formation which pose significant challenge for treatment [19, 20]. Because *B. henselae* is extremely fastidious, it is difficult to isolate and culture in liquid media especially from clinical samples. Therefore, the diagnosis is often combined with clinical features, serology, and PCR instead of culture to confirm [21]. Treatment of systemic *B. henselae* infections has been difficult with poor clinical outcomes despite antibiotic treatment for weeks and months [22].

To identify agents that are useful for treating persistent *B. henselae* infection, in our previous studies, we have used the SYBR Green/PI viability assay for drug screens against stationary phase *B. henselae* successfully [23–26]. In this study, we used the same SYBR Green/PI methodology and evaluated a range of commonly used antibiotics and agent from our recent screen [26] and their combinations. We identified several drug candidates and drug combinations that have much better activity against stationary phase and *B. henselae* biofilms. Azithromycin and rifampin are typically used as the first-line treatment for local manifestations of *Bartonella* infections, and doxycycline and gentamicin are used to treat trench fever, chronic bacteremia and endocarditis [26]. Often, with serious infections, more than one antibiotic is used. Thus, in our study, we also evaluated the efficiency of azithromycin or rifampin plus other antibiotics against stationary phase and *B. henselae* biofilms. Our study was the first to evaluate drug combinations against *B. henselae* non-growing stationary phase bacteria and biofilms and could provide experimental basis for further clinical evaluation.

## Results

### Growth behavior of B. henselae in modified Schneider’s medium

**Fig. 1:**
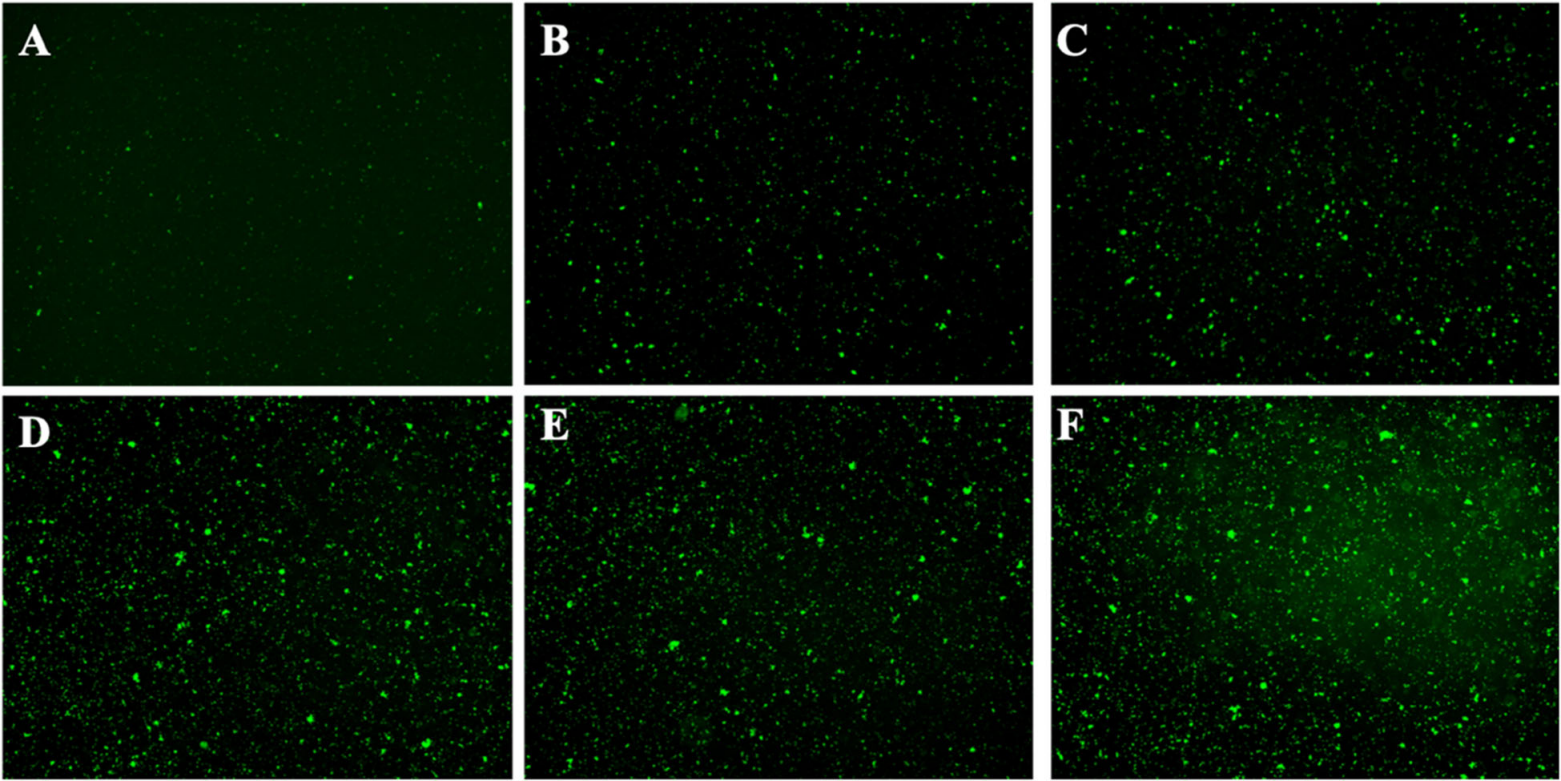
Representative images of 1 day (A), 2 day (B), 3 day (C), 4 day (D), 5 day (E) and 6 day (F) old *B. henselae* cultures. The *Bartonella henselae* cultures of varying ages were stained with SYBR Green I/PI assay and observed under the fluorescence microscope (400 × magnification). The bacterial cells were stained as green by SYBR Green I

The *B. henselae* cultures of varying ages (1 day, 2 day, 3 day, 4 day, 5 day and 6 day) were stained with SYBR Green I/PI assay and observed under the microscope (400 × magnification). The initial inoculum size was 1 × 10^6^ CFU/mL. As shown in Fig. 1, *B. henselae* grew to logarithmic growth phase in 1 to 2 days, and then reached stationary phase from 3 to 6 days. This is consistent with the bacterial growth curve reported in our previous study [26]. Based on these, we considered 1 to 2 day old *B. henselae* culture as log phase culture and 3 to 6 day old culture as stationary phase culture.

### MICs of candidate drugs

The candidate antibiotics evaluated were based on some antibiotics with good activity against stationary phase *B. henselae* [26] as well as antibiotics commonly used to treat *B. henselae* infections as controls. We used the standard method to determine the MICs of the candidate drugs for *B. henselae* after incubation of 6 days after drug addition as described in our previous study [26]. As shown in Table 1, rifampin was the most active agent which could inhibit *B. henselae* proliferation at the lowest concentration of <0.01 μg/mL. The growth of *B. henselae* was inhibited efficiently by azithromycin, doxycycline and methylene blue at a concentration of 0.08–0.16 μg/mL, by amikacin and nitrofurantoin at 0.31–0.63 μg/mL, by gentamicin at 0.63–1.25 μg/mL, by ciprofloxacin at 1.25–2.5 μg/mL, by cefuroxime, disulfiram, miconazole and SXT at 2.5–5.0 μg/mL. Daptomycin had relatively poor activity against growing *B. henselae* with a high MIC of 10–20 μg/mL. Clofazimine was the least effective agent for inhibiting the growth of *B. henselae*, with MIC higher than 40 μg/mL.

**Table 1:** MICs of select drug candidates against *B. henselae*

Antibiotics	MIC (μg/mL)
Amikacin	0.31–0.63
Azithromycin	0.08–0.16
Cefuroxime	2.5–5.0
Ciprofloxacin	1.25–2.5
Clofazimine	> 40
Daptomycin	10.0–20.0
Disulfiram	2.5–5.0
Doxycycline	0.08–0.16
Gentamicin	0.63–1.25
Methylene blue	0.08–0.16
Miconazole	2.5–5.0
Nitrofurantoin	0.31–0.63
Rifampin	< 0.01
SXT	2.5–5.0

### Drug exposure study to assess the activity of candidate drugs or drug combinations against stationary phase B. henselae

To confirm the activity of the drugs and drug combinations in killing stationary phase *B. henselae*, we performed a 24-h drug exposure study against a six-day-old stationary phase *B. henselae* culture as described in our previous study [26]. The concentration of each antibiotic was 5 μg/mL, as it is the average of most antibiotics’ Cmax in serum. As shown in Table 2, when used alone, nitrofurantoin, gentamicin, and ciprofloxacin were the most active agents, with 2.8 × 10^2^ CFU/mL, 5 × 10^2^ CFU/mL, and 6 × 10^2^ CFU/mL, surviving, respectively. Methylene blue and amikacin had significant activity with 10^4^ CFU/mL remaining. Rifampin, cefuroxime, azithromycin had moderate activity with 10^5–6^ CFU/mL remaining. Doxycycline, disulfiram, SXT had weak activity. In contrast, clofazimine and miconazole had poor activity against stationary phase *B. henselae*, with no obvious decrease in CFU compared with the drug-free control. In the two drug combination study, it is worth noting that among the 13 azithromycin drug combinations, only azithromycin/ciprofloxacin and azithromycin/methylene blue combinations were able to completely eradicate all stationary phase *B. henselae*, whereas 11 other azithromycin drug combinations were not able to do so (Table 2). Similarly, among the 12 rifampin two drug combinations, only rifampin/ciprofloxacin and rifampin/methylene blue were found to rapidly kill stationary phase *B. henselae* with no detectable CFU after 1-day exposure.

**Table 2:** Effect of drugs or drug combinations on survival of stationary phase *B. henselae*^a^

Drugs (5 μg/ml)	CFU per mL after drug exposure
Drug free control	2.8 ± 0.4 × 10^10^
Amikacin	8.0 ± 0.2 × 10^4^
Azithromycin	6.5 ± 0.4 × 10^5^
Cefuroxime	2.2 ± 0.2 × 10^5^
Ciprofloxacin	6.0 ± 0.1 × 10^2^
Clofazimine	1.6 ± 0.2 × 10^10^
Daptomycin	5.0 ± 0.2 × 10^7^
Disulfiram	1.0 ± 0.2 × 10^7^
Doxycycline	8.0 ± 0.3 × 10^6^
Gentamicin	5.0 ± 0.2 × 10^2^
Methylene blue	3.2 ± 0.4 × 10^4^
Miconazole	1.5 ± 0.1 × 10^10^
Nitrofurantoin	2.8 ± 0.1 × 10^2^
Rifampin	6.0 ± 0.3 × 10^5^
SXT	3.5 ± 0.2 × 10^6^
Azithromycin+Amikacin	4.3 ± 0.3 × 10^5^
Azithromycin+Rifampin	2.0 ± 0.2 × 10^5^
Azithromycin+Cefuroxime	5.2 ± 0.2 × 10^5^
Azithromycin+Ciprofloxacin	0
Azithromycin+Clofazimine	2.2 ± 0.3 × 10^6^
Azithromycin+Daptomycin	1.8 ± 0.1 × 10^5^
Azithromycin+Disulfiram	1.4 ± 0.2 × 10^5^
Azithromycin+Doxycycline	1.2 ± 0.3 × 10^6^
Azithromycin+Gentamicin	5.1 ± 0.3 × 10^4^
Azithromycin+Methylene blue	0
Azithromycin+Miconazole	1.3 ± 0.2 × 10^5^
Azithromycin+Nitrofurantoin	4.0 ± 0.3 × 10^5^
Azithromycin+SXT	8.5 ± 0.1 × 10^6^
Rifampin+ Amikacin	1.6 ± 0.1 × 10^5^
Rifampin+Cefuroxime	2.2 ± 0.1 × 10^4^
Rifampin+Ciprofloxacin	0
Rifampin+Clofazimine	2.8 ± 0.1 × 10^5^
Rifampin+Daptomycin	1.2 ± 0.1 × 10^6^
Rifampin+Disulfiram	8.5 ± 0.2 × 10^5^
Rifampin+Doxycycline	1.2 ± 0.1 × 10^6^
Rifampin+Gentamicin	1.6 ± 0.1 × 10^4^
Rifampin+Methylene blue	0
Rifampin+ Miconazole	8.0 ± 0.3 × 10^5^
Rifampin+Nitrofurantoin	1.2 ± 0.1 × 10^5^
Rifampin+SXT	1.6 ± 0.1 × 10^5^

^a^Stationary phase *B. henselae* (6-day old) cells were treated with 5 μg/ml drugs alone or drug combinations for 24 h when the survival of the bacteria was determined by CFU count after wash (see Methods for more details)

**Fig. 2:**
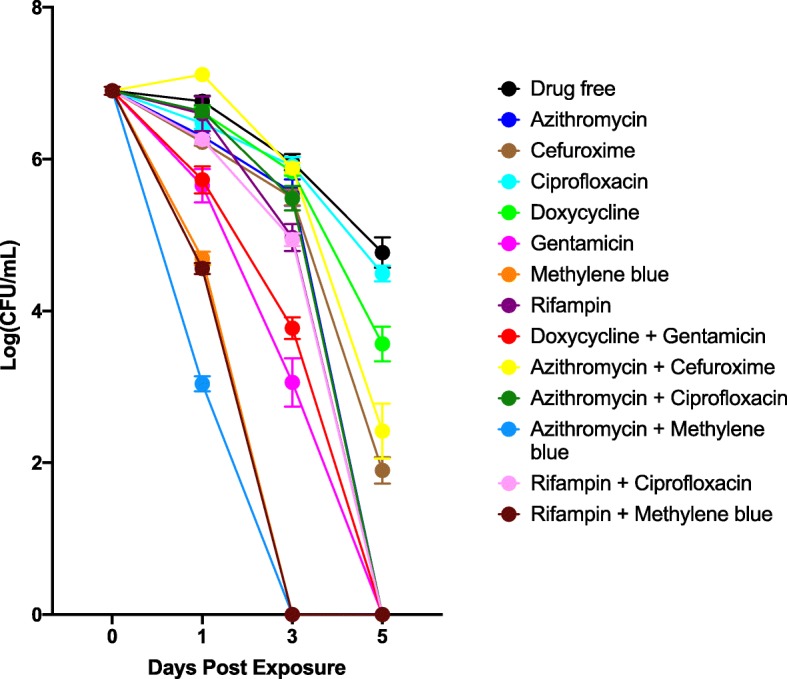
Time-kill curves of active drug combinations against six-day-old stationary phase *B. henselae* in comparison with clinical drugs. Antibiotics were added to the stationary phase culture at time point 0, and at different times of drug exposure (day 1, day 3, and day 5), portions of bacteria were removed and washed and plated on Columbia blood agar for CFU counts. The concentration of antibiotics was 5 μg/mL

In order to compare the efficacy of the identified active drug combinations with currently recommended antibiotic therapy (doxycycline/gentamicin) for treating B. henselae endocarditis, we performed a time-kill drug exposure assay of these active hits against a six-day-old stationary phase B. henselae culture. The concentration of each antibiotic was 5 μg/mL. As shown in Fig. 2, doxycycline/gentamicin could eradicate all stationary phase B. henselae cells after a 5-day drug exposure. Gentamicin was highly active even used alone, and methylene blue alone was more active than doxycycline/gentamicin combination. The drug combinations containing methylene blue, including azithromycin/methylene blue and rifampin/methylene blue were the most active ones that could rapidly kill all stationary phase B. henselae cells after a shorter time of 3-day drug exposure (Fig. 2), indicating our new drug combinations are more active than the currently recommended treatment with doxycycline/gentamicin combination.

### Biofilm formation in B. henselae culture

**Fig. 3:**
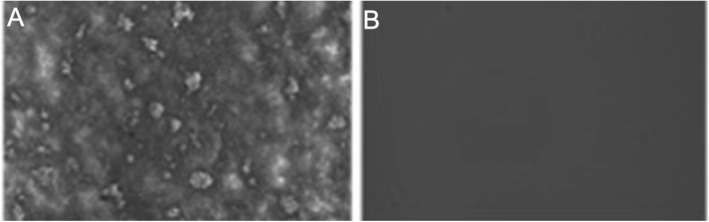
Representative images of *B. henselae* biofilm. (A) biofilm stained with crystal violet and observed under the microscope (400 × magnification) (B) negative control without *B. henselae* observed under the microscope (400 × magnification)

*B. henselae* was cultured in Schneider’s liquid medium at 37 °C, 5% CO_2_ for 5 days followed by dilution of the culture 1:100 into fresh Schneider’s medium for biofilm assays in 96-well plates for 5 days. The supernatant was carefully aspirated to prevent biofilm disruption, and then the biofilm was resuspended in Schneider’s medium and scraped up with a pipette tip. We found that compared with the control group, the bottom of the well could be seen to form a thin layer of biofilm with the naked eyes. Further examination under the microscope showed more obvious biofilm, as shown by aggregated structures of *B. henselae* cells (Fig. 3a) compared with negative control (Fig. 3b).

### Effect of select candidate drugs and drug combinations against biofilm recovered cells of B. henselae after drug exposure for different times

Since *B. henselae* biofilm contributes to its ability to persist in the host and could cause infective endocarditis that is difficult treat, it is important to eradicate the biofilm *B. henselae*. Based on the above results (Table 2), to further evaluate drug candidates against the biofilm *B. henselae* culture, we tested the efficacy of 12 antibiotics (azithromycin, cefuroxime, ciprofloxacin, daptomycin, disulfiram, doxycycline, gentamicin, methylene blue, miconazole, nitrofurantoin, rifampin, SXT) and the best 4 two-antibiotic combinations (azithromycin/ciprofloxacin, azithromycin/methylene blue, rifampin/ciprofloxacin, rifampin/methylene blue) in the biofilm *B. henselae* model after treatment for different times (2-day, 4-day, 6-day). The results are presented in Table 3. Overall, as expected, the biofilm derived cells of *B. henselae* were more tolerant to different drugs and drug combinations than the stationary phase cells (Table 2). No single drugs could completely eradicate all viable cells in the biofilm after drug treatment for 2 days, 4 days or 6 days (Table 3). Consistent with the results for stationary phase *B. henselae* drug exposure experiment, ciprofloxacin and gentamicin had good activity against biofilm *B. henselae* after 6 days of drug exposure, with 5.1 × 10^2^ CFU/mL and 8.1 × 10^2^ CFU/mL remaining, respectively (Table 3). Methylene blue and rifampin were the most active agents against the biofilm *B. henselae* after 6 days of drug exposure, with 2.3 × 10^2^ CFU/mL and 3.2 × 10^2^ CFU/mL, respectively (Table 3). Although nitrofurantoin was the most active agent against stationary phase *B. henselae*, its ability to kill biofilm *B. henselae* was poor after 6 days of drug exposure, with 2.8 × 10^9^ CFU/mL.

**Table 3:** Evaluation of select drug candidates against *B. henselae* biofilm-recovered cells after drug exposure at different times

Drugs (5 μg/ml)	CFU per mL after drug exposure		
	2 day	4 day	6 day
Drug free control	1.3 ± 0.2 × 10^10^	1.8 ± 0.2 × 10^10^	2.6 ± 0.3 × 10^10^
Azithromycin	4.5 ± 0.3 × 10^9^	5.2 ± 0.3 × 10^9^	9.3 ± 0.2 × 10^5^
Cefuroxime	5.6 ± 0.3 × 10^9^	2.3 ± 0.2 × 10^9^	1.7 ± 0.1 × 10^6^
Ciprofloxacin	3.2 ± 0.3 × 10^8^	2.5 ± 0.3 × 10^8^	5.1 ± 0.3 × 10^2^
Daptomycin	9.8 ± 0.2 × 10^9^	3.4 ± 0.3 × 10^9^	2.7 ± 0.2 × 10^6^
Disulfiram	6.1 ± 0.3 × 10^9^	4.6 ± 0.3 × 10^9^	3.8 ± 0.3 × 10^7^
Doxycycline	5.3 ± 0.3 × 10^9^	3.8 ± 0.1 × 10^9^	6.2 ± 0.3 × 10^5^
Gentamicin	6.2 ± 0.3 × 10^9^	5.8 ± 0.2 × 10^9^	8.1 ± 0.3 × 10^2^
Methylene blue	8.9 ± 0.4 × 10^9^	6.8 ± 0.2 × 10^9^	2.3 ± 0.2 × 10^2^
Miconazole	9.8 ± 0.3 × 10^9^	2.2 ± 0.1 × 10^10^	1.6 ± 0.1 × 10^10^
Nitrofurantoin	4.3 ± 0.1 × 10^9^	3.6 ± 0.2 × 10^9^	2.8 ± 0.2 × 10^9^
Rifampin	5.6 ± 0.2 × 10^9^	4.8 ± 0.3 × 10^9^	3.2 ± 0.2 × 10^2^
SXT	7.8 ± 0.3 × 10^9^	6.4 ± 0.3 × 10^9^	2.7 ± 0.2 × 10^9^
Azithromycin+Ciprofloxacin	4.1 ± 0.2 × 10^9^	5.8 ± 0.3 × 10^6^	0
Azithromycin+Methylene blue	6.8 ± 0.2 × 10^9^	5.2 ± 0.3 × 10^5^	0
Rifampin+Ciprofloxacin	7.6 ± 0.2 × 10^9^	4.7 ± 0.2 × 10^9^	0
Rifampin+Methylene blue	5.9 ± 0.1 × 10^9^	4.8 ± 0.2 × 10^9^	0

In the drug combination study, the 4 two drug combinations (azithromycin/ciprofloxacin, azithromycin/methylene blue, rifampin/ciprofloxacin, rifampin/methylene blue) had little activity against the biofilm bacteria after 2 day drug exposure with more than 10^9^ bacteria remaining. Interestingly, after 4 day drug exposure, azithromycin/ciprofloxacin, azithromycin/methylene blue were more effective than rifampin/ciprofloxacin, rifampin/methylene blue as the former azithromycin drug combinations had 10^5–6^ bacteria remaining while the rifampin drug combination still had a very high CFU count of 10^9^ bacteria. Nevertheless, all the 4 two drug combinations (azithromycin/ciprofloxacin, azithromycin/methylene blue, rifampin/ciprofloxacin, rifampin/methylene blue) completely eradicated all biofilm bacteria with no viable bacteria detected after 6 day drug exposure (Table 3).

## Discussion

In this study, to develop more effective treatment for persistent *Bartonella* infections, we mainly focused on evaluating drugs and drug combinations for activity against stationary phase and biofilm *B. henselae*. We found that in single drug treatments, ciprofloxacin, gentamicin, and nitrofurantoin were the most active agents against stationary phase *B. henselae*, and methylene blue and rifampin were the most active agents against the biofilm *B. henselae*. In drug combination studies, none of the two drug combinations were able to completely kill the biofilm bacteria in drug exposure up to 4 days, but the two azithromycin drug combinations (azithromycin/ciprofloxacin, azithromycin/methylene blue) seemed to be more active than rifampin drug combinations (rifampin/ciprofloxacin, rifampin/methylene blue) at Day 4. Interestingly, all four two drug combinations (azithromycin/ciprofloxacin, azithromycin/methylene blue, rifampin/ciprofloxacin, rifampin/methylene blue) could rapidly kill stationary phase and biofilm *B. henselae*.

Ciprofloxacin is a second-generation fluoroquinolone with a broad spectrum of activity that usually results in the killing of the bacteria. It is active against some Gram-positive and many Gram-negative bacteria including bacterial pathogens responsible for community-acquired pneumonias, bronchitis, urinary tract infections, and gastroenteritis [27]. Ciprofloxacin functions by inhibiting DNA gyrase, a type II topoisomerase, and topoisomerase IV, necessary to separate bacterial DNA, thereby inhibiting cell division [28, 29]. It has been reported that ciprofloxacin can be used in CSD [8, 30].

In our previous study, we found that methylene blue has good activity against stationary phase *B. henselae* [26]. Methylene blue is a medication and dye. As a medication, it is mainly used to treat methemoglobinemia [31]. It is also used as an antimalarial agent and for urinary tract infection (UTIs) treatment [32]. Recent studies found that methylene blue had antifungal effect through redox and membrane disruption [33]. While membrane is a target of persister drugs, our previous finding that methylene blue also had activity against *Borrelia burgdorferi* stationary phase cells is consistent with these [25]. It remains to be determined whether methylene blue could disrupt membranes of *B. henselae* as its basis for killing non-growing stationary phase *B. henselae* in the future.

For persistent and severe infections such as *B. henselae* infections, one drug is not enough, and a drug combination approach is needed [34, 35]. In our study, we evaluated 25 two-antibiotic combinations for activity against stationary phase *B. henselae*. We found four two-antibiotic combinations (azithromycin/ciprofloxacin, azithromycin/methylene blue, rifampin/ciprofloxacin, rifampin/methylene blue) had good activity against stationary phase *B. henselae* with no colony being detected. Furthermore, we evaluated the ability of the 4 two-antibiotic combinations against the biofilm of *B. henselae* and found that in general biofilm bacteria are more difficult to eradicate and it took longer (6 days) for the antibiotic combinations to eradicate the biofilm bacteria (Table 3) than the stationary phase bacteria (1 day) (Table 2). However, it is worth noting that azithromycin/ciprofloxacin and azithromycin/methylene blue were more active than the rifampin/ciprofloxacin and rifampin/methylene blue combinations. For convenience, the drug concentration of 5 μg/mL used in drug combination studies was based on average of Cmax for most antibiotics. The exact Cmax concentrations for each promising drug combination can be used in future drug combination studies.

Because azithromycin and rifampin are the first line drugs for treating *B. henselae* infections, our study only evaluated the efficiency of some two-drug combinations which we found to be more active than single drugs alone. In the future, we could try more three drug combinations using the current drugs used in clinic with the newly identified drug candidates to kill different bacterial populations for more effective eradication, especially focusing on oral drugs. The biofilm model we used was 5 day old and scraped off the 96-well plate and could be considered young biofilm and may be more easily killed than older and intact biofilms. Future studies are needed to evaluate promising drug combinations on the latter more difficult to kill intact biofilms. It is worth noting that the anti-*Bartonella* activity of these identified drug combinations were obtained from in vitro assay, and further pharmacokinetic studies and in vivo animal efficacy studies are needed. If animal study results are favorable, clinical trials can be conducted to assess the safety and efficacy of the identified active drug combinations. Finally, we need to determine whether our findings derived from one strain *B. henslelae* JK53 are valid for other *B. henselae* strains and other pathogenic *Bartonella* species, such as *B. quintana* and *B. bacilliformis*.

## Conclusions

This study identified methylene blue, gentamicin, and nitrofurantoin among 14 antibiotics evaluated to be the most active agents against stationary phase *B. henselae*, and drug combinations azithromycin/ciprofloxacin, azithromycin/methylene blue, rifampin/ciprofloxacin, and rifampin/methylene blue could kill stationary phase and bioifilm *B. henselae* with no detectable CFU. Future studies are needed to confirm the activity of the above active drugs or drug combinations in vivo animal and human studies to assess their utility to improve the treatment of persistent *Bartonella* infections.

## Methods

### Bacterial strain, culture media and culture conditions

*Bartonella henselae* strain JK53 was obtained through BEI Resources (ATCC), NIAID, NIH. *B. henselae* was cultured in Schneider’s medium supplemented with 10% fetal bovine serum (FBS) as described [26, 36]. Cultures were incubated at 37 °C, 5% CO_2_ at all times without shaking. The colony forming unit (CFU) counting was performed after serial dilutions on Columbia sheep blood agar (Becton Dickinson Biosciences, California, USA).

### Antibiotics and stocks

Antibiotics including amikacin, azithromycin, cefuroxime, ciprofloxacin, clofazimine, daptomycin, disulfiram, doxycycline, gentamicin, methylene blue, miconazole, nitrofurantoin, rifampin and trimethoprim/sulfamethoxazole (SXT), were purchased from Sigma & Aldrich and were dissolved in appropriate solvents [37] to form stock solutions. All the antibiotic stocks were filter-sterilized by 0.2 μm filter except the DMSO stocks.

### Microscopy techniques

The SYBR Green I/propidium iodide (PI) dye was added to *B. henselae* cell suspensions for observing the growth of *B. henselae* as described previously [23, 24]. The strain samples were examined on a BZ-X710 All-in-One fluorescence microscope (KEYENCE, Inc.). The biofilm specimens were stained with 0.1% crystal violet and observed under the microscope (400 × magnification).

### Assessing drug activity against stationary phase B. henselae

Based on our previous study [26], we selected 14 antibiotics (amikacin, azithromycin, cefuroxime, ciprofloxacin, clofazimine, daptomycin, disulfiram, doxycycline, gentamicin, methylene blue, miconazole, nitrofurantoin, rifampin, SXT) and 25 two antibiotics combinations (azithromycin/amikacin, azithromycin/rifampin, azithromycin/cefuroxime, azithromycin/ciprofloxacin, azithromycin/clofazimine, azithromycin/daptomycin, azithromycin/disulfiram, azithromycin/doxycycline, azithromycin/gentamicin, azithromycin/methylene blue, azithromycin/miconazole, azithromycin/nitrofurantoin, azithromycin/SXT, rifampin/amikacin, rifampin/cefuroxime, rifampin/ciprofloxacin, rifampin/clofazimine, rifampin/daptomycin, rifampin/disulfiram, rifampin/doxycycline, rifampin/gentamicin, rifampin/methylene blue, rifampin/miconazole, rifampin/nitrofurantoin, rifampin/SXT) for drug screen against stationary phase *B. henselae*. 100 μL *B. henselae* cell suspension from a 6-day old stationary phase culture was added in 96-well plates. Each compound (10 μL, each antibiotic’s final concentration was 5 μg/ml, including antibiotic combination) from the pre-diluted plate or pre-diluted stock was added to the cell suspension. Plates were sealed and placed in 37 °C incubator for 5 days. After antibiotic exposure, SYBR Green I/ PI viability assay was used to assess the live and dead cells as described [26]. 10 μL SYBR Green I (100× stock, Invitrogen, Waltham, MA, USA) and propidium iodide (PI, 600 μM, Sigma, St. Louis MO, USA) staining mixture was added to each well and mixed thoroughly. The plates were incubated in the dark for 15 min at room temperature, and then read using microplate reader (HTS 7000 plus Bioassay Reader, Perkin Elmer Inc., Waltham MA, USA). The green/red (535 nm/635 nm) fluorescence ratio of each well was used for calculating the residual viability percentage with least-square fitting analysis as described previously [26]. All tests were run in triplicate.

### MIC determination

To determine the minimum inhibitory concentration (MIC) needed to inhibit visible growth of *B. henselae* after 6-day incubation using the standard microdilution method. *B. henselae* cells (1 × 10^6^) from a 6 day old stationary phase culture were inoculated with 90 μL fresh modified Schneider’s medium into each well of 96-well microplate. Each diluted drug (10 μL) was then added to the culture. The 96-well plates were sealed and incubated at 37 °C with 5% CO_2_ for 5 days. After the incubation, cell proliferation was assessed using the SYBR Green I/PI assay and a Petroff-Hausser counting chamber. All experiments were run in triplicate.

### Drug exposure assay for stationary phase B. henselae

A six-day-old *B. henselae* stationary phase culture was used for drug exposure experiments. The antibiotic exposure was carried out for 24 h or 5 days at 37 °C without shaking in 1.5 mL Eppendorf tubes. The concentration of each antibiotic was 5 μg/mL. Then the culture was centrifugated to collect the cells, and rinsed with fresh Schneider’s medium twice, and then resuspended in 1 mL fresh Schneider’s medium. The cell suspension was serially diluted and plated on Columbia blood agar plates for viable bacterial counts (colony forming unit, CFU).

### Drug exposure assay for B. henselae biofilm

For biofilm inoculum, *B. henselae* was cultured in Schneider’s liquid medium at 37 °C, 5% CO_2_ for 5 days. The culture was diluted 1:100 into fresh Schneider’s medium for biofilm assays in 96-well plates for 5 days. The supernatant was carefully aspirated to prevent biofilm disruption, and then resuspended in Schneider’s medium and scraped up with a pipette tip. The biofilm was stained as described previously [38]. The antibiotic exposure was carried out as described above, except the drug exposure was 2 days, 4 days and 6 days.

## Data Availability

Not applicable.

## Abbreviations

CFU: Colony forming unit
CSD: Cat scratch disease
DMSO: Dimethyl sulfoxide
FBS: Fetal bovine serum
MIC: Minimum inhibitory concentration
PBS: Phosphate-buffered saline
PI: Propidium iodide
SXT: Sulfamethoxazole/Trimethoprim

## References

[1] Gutierrez R, Vayssier-Taussat M, Buffet JP, Harrus S (2017). Guidelines for the isolation, molecular detection, and characterization of Bartonella species. Vector Borne Zoonotic Dis.

[2] Deng H, Pang Q, Zhao B, Vayssier-Taussat M (2018). Molecular mechanisms of Bartonella and mammalian erythrocyte interactions: a review. Front Cell Infect Microbiol.

[3] Rolain JM, La Scola B, Liang Z, Davoust B, Raoult D (2001). Immunofluorescent detection of intraerythrocytic Bartonella henselae in naturally infected cats. J Clin Microbiol.

[4] Deng H, Le Rhun D, Buffet JP, Cotte V, Read A, Birtles RJ, Vayssier-Taussat M (2012). Strategies of exploitation of mammalian reservoirs by Bartonella species. Vet Res.

[5] Mullins KE, Hang J, Clifford RJ, Onmus-Leone F, Yang Y, Jiang J, Leguia M, Kasper MR, Maguina C, Lesho EP (2017). Whole-genome analysis of Bartonella ancashensis, a novel pathogen causing Verruga Peruana, rural Ancash region, Peru. Emerg Infect Dis.

[6] Karem KL, Paddock CD, Regnery RL (2000). Bartonella henselae, B. quintana, and B. bacilliformis: historical pathogens of emerging significance. Microbes Infect.

[7] Yuan C, Zhu C, Wu Y, Pan X, Hua X (2011). Bacteriological and molecular identification of Bartonella species in cats from different regions of China. PLoS Negl Trop Dis.

[8] Hamilton DH, Zangwill KM, Hadler JL, Cartter ML (1995). Cat-scratch disease--Connecticut, 1992-1993. J Infect Dis.

[9] Chomel BB, Kasten RW, Floyd-Hawkins K, Chi B, Yamamoto K, Roberts-Wilson J, Gurfield AN, Abbott RC, Pedersen NC, Koehler JE (1996). Experimental transmission of Bartonella henselae by the cat flea. J Clin Microbiol.

[10] Debre R (1950). Cat scratch disease. Mars Med.

[11] Jackson LA, Perkins BA, Wenger JD (1993). Cat scratch disease in the United States: an analysis of three national databases. Am J Public Health.

[12] Pulliainen AT, Dehio C (2012). Persistence of Bartonella spp. stealth pathogens: from subclinical infections to vasoproliferative tumor formation. FEMS Microbiol Rev.

[13] Deng H, Pang Q, Xia H, Le Rhun D, Le Naour E, Yang C, Vayssier-Taussat M, Zhao B (2016). Identification and functional analysis of invasion associated locus B (IalB) in Bartonella species. Microb Pathog.

[14] Okaro U, Addisu A, Casanas B, Anderson B (2017). Bartonella species, an emerging cause of blood-culture-negative endocarditis. Clin Microbiol Rev.

[15] Chomel BB, Boulouis HJ, Breitschwerdt EB, Kasten RW, Vayssier-Taussat M, Birtles RJ, Koehler JE, Dehio C (2009). Ecological fitness and strategies of adaptation of Bartonella species to their hosts and vectors. Vet Res.

[16] Bjarnsholt T. The role of bacterial biofilms in chronic infections. *APMIS Suppl*. 2013:1–51. 10.1111/apm.12099. 10.1111/apm.12099 23635385

[17] Okshevsky M, Meyer RL (2015). The role of extracellular DNA in the establishment, maintenance and perpetuation of bacterial biofilms. Crit Rev Microbiol.

[18] Rolain, J.M.; Brouqui, P.; Koehler, J.E.; Maguina, C.; Dolan, M.J.; Raoult, D. Recommendations for treatment of human infections caused by Bartonella species. Antimicrob Agents Chemother 2004, 48, 1921-1933, doi:10.1128/AAC.48.6.1921-1933.2004. 10.1128/AAC.48.6.1921-1933.2004 PMC415619 15155180

[19] Schulein R, Seubert A, Gille C, Lanz C, Hansmann Y, Piemont Y, Dehio C (2001). Invasion and persistent intracellular colonization of erythrocytes. A unique parasitic strategy of the emerging pathogen Bartonella. J Exp Med.

[20] Breitschwerdt EB, Maggi RG, Lantos PM, Woods CW, Hegarty BC, Bradley JM (2010). Bartonella vinsonii subsp. berkhoffii and Bartonella henselae in a father and daughter with neurological disease. Parasit Vectors.

[21] Chomel BB, Boulouis HJ, Maruyama S, Breitschwerdt EB (2006). Bartonella spp. in pets and effect on human health. Emerg Infect Dis.

[22] Angelakis E, Raoult D (2014). Pathogenicity and treatment of Bartonella infections. Int J Antimicrob Agents.

[23] Feng J, Wang T, Zhang S, Shi W, Zhang Y (2014). An optimized SYBR green I/PI assay for rapid viability assessment and antibiotic susceptibility testing for Borrelia burgdorferi. PLoS One.

[24] Feng J, Wang T, Shi W, Zhang S, Sullivan D, Auwaerter PG, Zhang Y (2014). Identification of novel activity against Borrelia burgdorferi persisters using an FDA approved drug library. Emerg Microbes Infect.

[25] Feng J, Weitner M, Shi W, Zhang S, Sullivan D, Zhang Y (2015). Identification of additional anti-Persister activity against Borrelia burgdorferi from an FDA drug library. Antibiotics (Basel).

[26] Li T, Feng J, Xiao S, Shi W, Sullivan D. Identification of FDA-Approved Drugs with Activity against Stationary Phase Bartonella henselae. Antibiotics (Basel). Zhang, Y, 2019:8. 10.3390/antibiotics8020050. 10.3390/antibiotics8020050 PMC6628006 31035691

[27] Bennett AC, Bennett CL, Witherspoon BJ, Knopf KB. An evaluation of reports of ciprofloxacin, levofloxacin, and moxifloxacin-association neuropsychiatric toxicities, long-term disability, and aortic aneurysms/dissections disseminated by the Food and Drug Administration and the European Medicines Agency. *Expert Opin Drug Saf*. 2019:1–9. 10.1080/14740338.2019.1665022. 10.1080/14740338.2019.1665022 PMC9830651 31500468

[28] Drlica K, Zhao X (1997). DNA gyrase, topoisomerase IV, and the 4-quinolones. Microbiol Mol Biol Rev.

[29] Jin H, Qi C, Zou Y, Kong Y, Ruan Z, Ding H, Xie X, Zhang J (2017). Biochanin a partially restores the activity of ofloxacin and ciprofloxacin against topoisomerase IV mutation-associated fluoroquinolone-resistant Ureaplasma species. J Med Microbiol.

[30] Windsor JJ (2001). Cat-scratch disease: epidemiology, aetiology and treatment. Br J Biomed Sci.

[31] Yamaji F, Soeda A, Shibata H, Morikawa T, Suzuki K, Yoshida S, Ogura S (2018). A new mutation of congenital methemoglobinemia exacerbated after methylene blue treatment. Acute Med Surg.

[32] Schirmer RH, Adler H, Pickhardt M, Mandelkow E (2011). Lest we forget you--methylene blue. Neurobiol Aging.

[33] Ansari MA, Fatima Z, Hameed S (2016). Antifungal action of methylene blue involves mitochondrial dysfunction and disruption of redox and membrane homeostasis in C. albicans. Open Microbiol J.

[34] Zhang Y (2014). Persisters, persistent infections and the yin-Yang model. Emerg Microbes Infect.

[35] Feng J, Weitner M, Shi W, Zhang S, Zhang Y (2016). Eradication of biofilm-like microcolony structures of Borrelia burgdorferi by Daunomycin and Daptomycin but not Mitomycin C in combination with doxycycline and cefuroxime. Front Microbiol.

[36] Riess T, Dietrich F, Schmidt KV, Kaiser PO, Schwarz H, Schäfer A, Kempf VA (2008). Analysis of a novel insect cell culture medium-based growth medium for Bartonella species. Appl Environ Microbiol.

[37] Clinical and Laboratory Standards Institute. Performance Standards for Antimicrobial Susceptibility Testing; Seventeenth Informational Supplement. *CLSI document M100-S17*, 2007. *27*:154–61.

[38] O'Toole GA. Microtiter dish biofilm formation assay. J Vis Exp. 2011. 10.3791/2437. 10.3791/2437 PMC3182663 21307833

